# What the General Practitioner Should Know about Diseases of the Eye

**Published:** 1893-08

**Authors:** Frank Trester Smith

**Affiliations:** Professor of Diseases of the Eye, Chattanooga Medical College, Chattanooga, Tenn.


### WHAT THE GENERAL PRACTITIONER SHOULD KNOW ABOUT DISEASES OF THE EYE.*

BY FRANK TRESTER SMITH, A. M., M. D.,
Professor of Diseases of the Eye, Chattanooga Medical College,
CHATTANOOGA, TENN.

That there is a lack of knowledge in the profession regarding the elementary principles of ophthalmology will hardly be denied by any one who is at all familiar with the facts. Our crowded college courses, and the feeling among students that they are not required in actual practice to know anything of the eye, owing to the multiplication of specialists, are largely responsible for this. Again, the subject is so extensive that it is difficult to decide where to leave off the study, and instead of mastering a few general principles with the more important facts, too often the matter is skimmed over and a superficial knowledge of much is obtained instead of a thorough acquaintance with what is most necessary. It will be the object of this paper to define the minimum amount of knowledge

*Read before the Tennessee State Medical Society.

of this subject which one should have before entering the practice of medicine. If he understands these things well he will find it much better for his reputation, his pocketbook and for his patient; for if he makes a mistake elsewhere the kindly earth will cover it up, but here an error will be a walking advertisement to his want of skill or lack of knowledge.

In the first place, it is not to be expected that every doctor should become a specialist or in any sense competent to deal with all eye affections, but he should know enough to know his ignorance. Secondly, it is not necessary for him to understand how to fit glasses, but he should know the main indications for the use of spectacles, *e. g.*, presbyopia, diminished vision, asthenopia, headache, functional nervous disorders. In the third place, he should not attempt to use the ophthalmoscope for several reasons. First, it will not pay. He will not realize enough out of these cases to pay the cost of a good instrument. Then there is the cost and time of learning. It would be cheaper for him to pay a fee out of his own pocket to a competent specialist. He could not keep in practice for this work. Most books on diseases of the eye have a chapter on the ophthalmoscope which always seemed to me as worse than useless.

We can divide the cases under consideration into four classes: 1, Those with impaired vision; 2, those complaining of pain referred to the eye; 3, the inflammatory cases; 4, the miscellaneous cases. Your patrons have a right to demand that you know something of these and not depend entirely on the oculist. You will be compelled to give advice in all these cases, and you should know how to advise and when it is necessary to call in a specialist. You should know that every case of impaired vision and any clear cornea should have the benefit of an expert examination, provided that there is any perception of light. The doctor should be able to test for perception of light. He should know that where there is no perception of light, or where the cornea is opaque, that it is not worth while to subject your patron to any expense for an examination. He should know that where spectacles are supposed to be required that the oculist, and not the traveling charlatan nor the optician, is the proper person to whom these cases should be referred. He should be able to diagnose between glaucoma and cataract as a cause for the poor vision, and not advise the former to wait until it gets ripe, with the result that a specialist is consulted when it is too late to preserve any vision.

In our second class of cases, in which pain is the prominent symptom, glaucoma is at once suggested. With the diagnosis of this disease every practitioner should be familiar. It does not require any skill to diagnose increased tension (hardness) of the eyeball in a well-marked case, and the other points of the diagnosis should be well understood. Pain in the eye without inflammatory symptoms is more often glaucoma than neuralgia. In some cases of glaucoma the reflex symptoms have predominated so that cases have been treated for biliousness. You should know enough to prescribe myotics until an operation can be performed.

The general practitioner will be most often consulted in reference to our third class of cases, the inflammations. In all inflammations of the eye atropine is indicated, with the exception of the inflammations of the conjunctiva. Even here it will, as a rule, do no harm, so that in a case of doubt it would be best to use it, as the neglect of this agent may result disastrously. But the doctor should know that it is to be used with caution in all cases over forty years, and that it is positively contraindicated in cases where there is any tendency to glaucoma. While many inflammations of the eye tend to spontaneous cure, and others would get well if treated on the above general principles, still it would be better to consult a specialist early and carry out a line of treatment laid down by him than to send him the case after all other resources are exhausted. The doctor should know the indications and the contra-indications of atropine and also of aserine.

In some cases it is difficult to diagnose between glaucoma and serous iritis. If it were iritis and aserine or pilocarpine used to contract the pupil, the case would be made worse. If it were glaucoma and atropine or other mydriatic used, the result would be disastrous.

Under the fourth head are included all cases of functional nervous disorders, reflex and otherwise, which may be benefited by wearing glasses, or by some treatment, operative or otherwise, of the eyes. This includes cases of asthenopia, headache, chorea, epilepsy and all obscure nervous troubles. It is sufficient for you to remember that many of these cases have been relieved and some cured by treating their eyes.

Injuries of the eyeball should, as a rule, be referred to a specialist at an early date, and especially if there is a suspicion of a foreign body, on account of the danger of sympathetic ophthalmia.

An endeavor has here been made to briefly outline the minimum knowledge of this branch which a general practitioner should have. More might be useful. For instance, the size and motility of the pupil will often aid in diagnosing cerebral affections or general diseases.

What has been given seems to be indispensable to any one who wants to practice medicine conscientiously.

